# The Scottish Simulation ‘KSDP’ Design Framework: a sense-making and ordered approach for building aligned simulation programmes

**DOI:** 10.1186/s41077-024-00321-3

**Published:** 2024-12-27

**Authors:** Nathan Oliver, Simon Edgar, Edward Mellanby, Alistair May

**Affiliations:** 1 University of Canberra, Canberra, Australia; 2 Scottish Centre for Simulation and Clinical Human Factors, Falkirk, Scotland; 3 Medical Education Directorate, NHS Lothian, Edinburgh, Scotland

**Keywords:** Simulation design, Programme design, Healthcare simulation

## Abstract

Impactful learning through simulation-based education involves effective planning and design. This can be a complex process requiring educators to master a varied toolkit of analysis tools, learning methodologies, and evaluative strategies; all to ensure engagement of learners in a meaningful and impactful way. Where there is a lack of thoughtful design, simulation-based education programmes may be inefficiently deployed at best, and completely ineffective or even harmful to learning and learners at worst. This paper presents a useful sense-making framework, designed to support simulation educators in designing their learning activities in a systematic, stepwise, and learner centred way.

Embedding simulation programmes within complex healthcare environments is challenging and often poorly executed, resulting in resistance from participants and a lack of tangible learning outcomes. Whilst significant work has already been achieved around the potential impact of a wide range of simulation modalities, there has been little conversation about the practicalities of embedding these in a structured and cohesive way within the ever-moving clinical environment. The development of a shared language and framework to support sense-making and sequencing stands to promote a radically different execution and impact experience for many simulation designers, of which to hang their simulation methodology from.

The process of designing simulation based procedural skills programmes [1–3], scenario design principles [4–6], structured debriefing [7–9], and the use of simulation informed systems testing [10–14] have been well described in the literature within their component parts. Roussin and Weinstock conceptualised a system for matching design decisions with appropriate delivery approaches through their ‘SimZones’ innovation [15]. ‘SimZones’ categorises the selection of an appropriate modality (‘do we require a manikin or a task trainer for this?’), for the learning need at hand (‘are we rehearsing psychomotor procedural skills or are we training team communication skills?’), in order to maximise impact [15]. That is all very well in theory, in the vacuum of the ideal atmospheric conditions of adequate funding, trained faculty, and abundant time. But that is not where we live. We live within the complexity of ‘real-world’ healthcare environments with a variable amount of these atmospheric conditions. What is desperately required for many clinical educators situated in clinical departments is:

- An approach that supports sense-making—what tool (or tools) are most appropriate in response to a dynamic set of learning needs.
- An approach that supports order-making—in which order and priority is most appropriate to meet that dynamic set of learning needs.

We present a theoretically informed approach to support programme design which is cognisant of both approaches. Developed in Scotland and utilised within the regional simulation network for over a decade, this framework promotes a shared language and way of thinking when sense-making and resource-designing simulation programmes, and effectively underpins a more strategic approach to simulation-based programmes.

## Limitations of designing simulation without an informed design

We know that the clinical environment is a highly complex and dynamic biosphere working 24 h a day. Patients and their families have complex needs. Resources stretched and staffing numbers are ever under pressure. Most activities are time-sensitive, invariably highly-emotive, and occasionally high-risk [16, 17]. This is a landscape many clinical educationalist readers will know all too well. Added to this, effective educational design is a complex process and requires a varied toolkit of analysis tools, learning methods, and evaluative strategies—depending on a variety of factors, to engage with learners in a meaningful and impactful way. Sometimes simulation will be a highly effective approach to meet the learning need, and sometimes it simply will not be. Other times simulation will be effective and efficient if designed in one way, however, be ineffective and inefficient if deployed in another. For every request therefore for ‘*more simulation*’, or in response to the question ‘*can you bring the expensive manikin to the ward for this?*’, the response is almost certainly ‘*it depends*’.

To illustrate this further, please consider this case study, which we will revisit later in the paper.

**Table:**
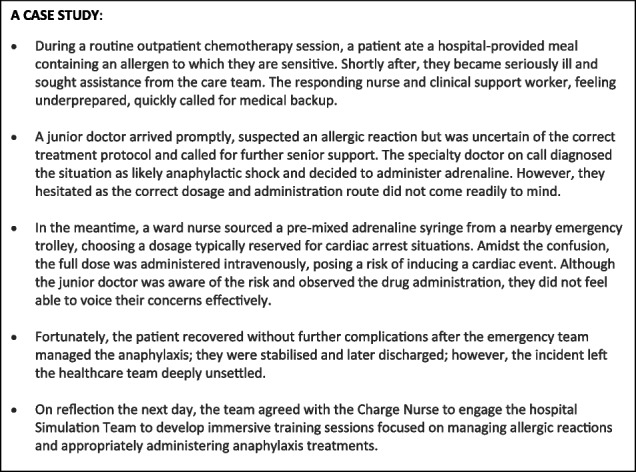

The difficulty in responding to this case study is threefold:

- **Psychological safety:** An immersive, performance-based simulation programme unexpectedly deployed is often received to clinical teams poorly who might easily experience it as a threat to their professional identity and as a punitive action [18].
- **Educational effectiveness:** An immersive, performance-based simulation programme may not be the most effective methodology to addressing the learning needs and gaps of the team. In fact, without thoughtful engagement with the underlying causes of the adverse event, it cannot be assumed that the primary issues are learning gaps at all.
- **Operational efficiency:** In the same way, deploying a technologically high end and resource and cost heavy intervention into a situation without a thoughtful framework is an extremely expensive and inefficient way to explore what may be a range of discrete issues within the system, which again, may or may not be all educational in nature [19].

We are convinced that simulation activity void of an informed platform of design will almost certainly be inefficient, will likely to be ineffective, and has real potential to be outright unsafe. We have seen the negative effects of an inappropriately designed simulation, where learners are emotionally scarred from their experiences, and vow never to engage with simulation activity again! In response to these challenges, we offer a framework which is simple and uncluttered yet affords its user a tool to cut through some of the features of its complexity and build a programme sequentially. Before doing so, we are first required to expound upon the underlying conceptual approaches to which it rests.

## Informing the design through sense-making and sequencing in a complex system

There are two conceptual approaches which are foundational to our framework: The Cynefin framework offers a lens to system navigators to aid decision-making in the face of increasing complexity, and cognitive load theory offers an approach to constructively build programmes of learning that maximally facilitate effective knowledge and skills acquisition.

### Sense-making (the drive for efficiency)

Sense-making here refers to the process of understanding one’s situational context and in doing so, create space for thoughtful decision-making under uncertainty [20]. It might be likened to a hiker pulling out her compass to make sense of her location and map her next steps. It is helpful in this section to explore what we mean by sense-making in the context of the complex clinical landscape. To support our discussion, we do so through the lens of the Cynefin framework [20]. Whilst the Cynefin framework has not been designed with education specifically in mind, no model is ‘a perfect fit’, and we assert (in agreement with others) that extending this sense-making approach to instructional design principles provides highly valuable insights [21].

**Fig. 1:**
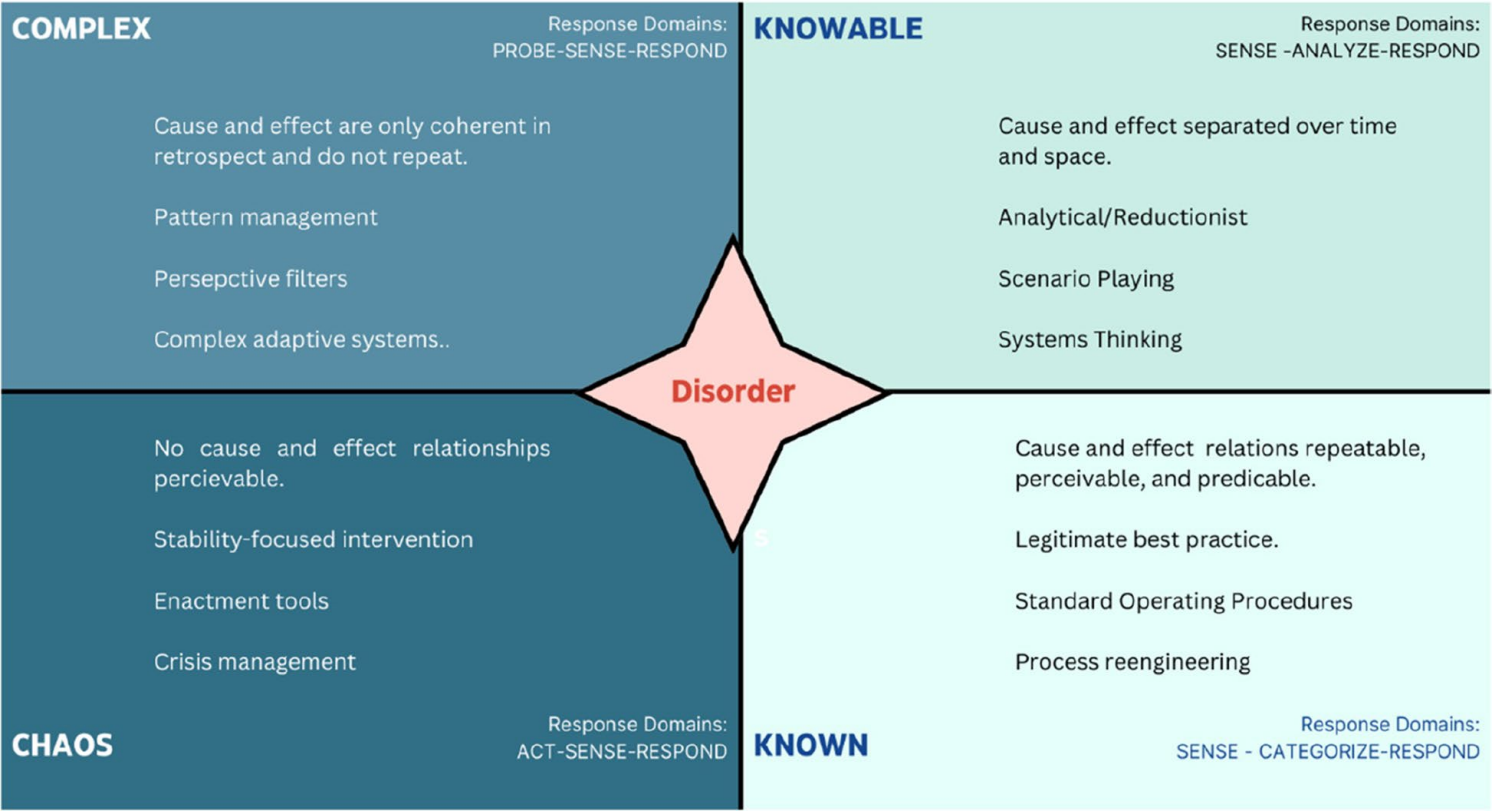
Cynefin framework [20]

The Cynefin framework, originating from the Welsh word for ‘domain’ or ‘habitat’, is a well-established sense-making model that supports knowledge management and decision-making across four distinct domains (see Fig. 1). This framework helps decision-makers by providing a structured approach to categorise and address issues based on their complexity. At the heart of Cynefin is the concept of ‘disorder’, represented centrally in the framework, where decision-makers often start without a clear context or understanding. According to Kurtz and Snowden, beginning from this state of disorder, a situation can be evaluated to determine which domain it fits into, facilitating appropriate responses [20].

For instance, if a decision-maker identifies a problem as well-known and straightforward, the framework directs action within the specific domain that corresponds to routine situations (illustrated in the lower right quadrant of the diagram). The approach involves understanding the problem, categorising it, and responding accordingly. Conversely, if the problem is chaotic and complex, the framework suggests a response that aligns with the chaotic domain (shown in the lower left quadrant), where actions are geared towards making sense of the disorder and responding dynamically; in the domain of systems improvement, these initial steps would be framed as quality planning and is better interrogated using translational simulation methodologies (at least initially), rather than simulation-based educational ones [10, 20].

**Fig. 2:**
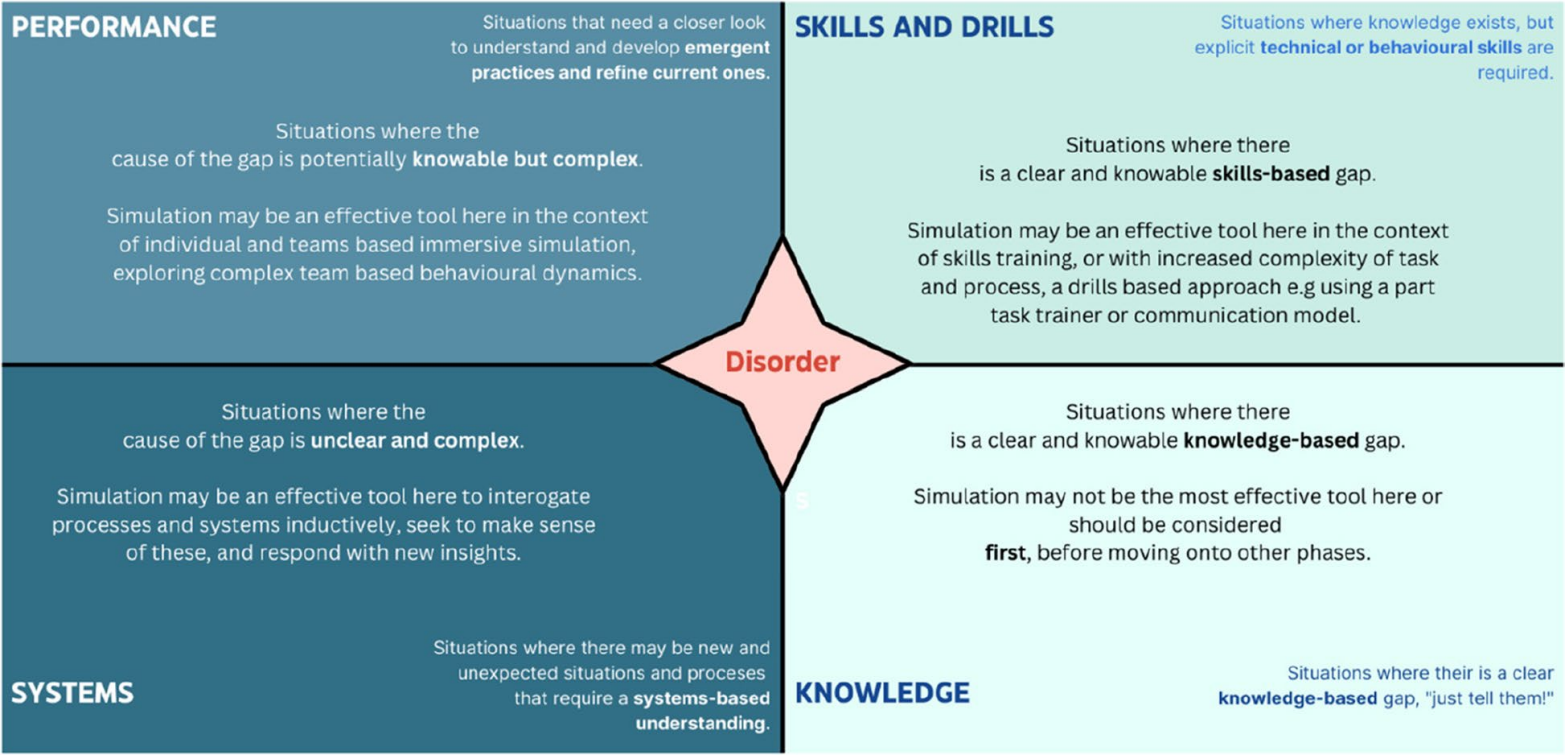
Cynefin framework (adapted) for simulation design

Figure 2 illustrates an adaptation of the Cynefin framework, tailored specifically for simulation-based educational design. Our adaptation emphasises sense-making capabilities, integrating a systems thinking approach to understand our complex health and care settings and address the unique needs of the staff or students who work in them. The scope of this paper narrows to the first three domains of the framework (known, knowable, complex), focusing on their application in designing learning and rehearsal activities within simulation settings.

Applying this adapted model to simulation-based pedagogy, we interpret ‘disorder’ as the initial phase of designing effective simulations in healthcare through a better understanding of the degree of complexity in real work.

Within the adapted framework (see Fig. 2), design decisions and created content are aligned with the domain that maximises impact efficiently. For example, simulation faculty often go to painstaking lengths to recreate the complex clinical environment for a simulation for learning needs that may be impactfully achieved in more efficient ways. If the primary learning gap is knowledge-based for instance, using an expensive mannequin may not be the most efficient choice; a journal article, tutorial, or lecture might suffice. Conversely, if the competence gap relates to psychomotor skills, these less tactile methods would be inadequate, whilst mannequin use and immersive scenarios could be excessive; a task trainer or modified mastery approach may be the design route of choice [3]. Therefore, efficient design hinges on a careful assessment of learner needs, navigating complexity initially and then building upon it with appropriate educational sequencing.

### Order-making (or the drive for effectiveness)

Where sense-making supports decision-making towards the approach (or approaches) that might be the most efficient in a complex environment, order-making supports decision-making towards a coherent and theoretically informed deployment of these. This can be illuminated through the lens of cognitive load theory (CTL), which looks at learning through the tradition of cognitive psychology with a focus on the interactions between working memory (WM) and long-term memory (LTM) [22, 23]. Simply put, humans have a large capacity within their LTM but a limited one within their WM. Information not processed quickly is quickly lost and is heavily influenced by intrinsic (for example, the level of difficulty the task is for the learner), extraneous (for example, the nature of the learning environment and the way it is presented), and germane loads (the inherent challenge of the task at hand) [22–24]. Where the level or type of extraneous load cannot be met by some previous knowledge or experience, a learner’s WM may become overwhelmed and not processed effectively into their LTM. Sequencing information in some kind of order, however, can help. For example, before getting behind the wheel of a car on a busy freeway, a learner driver will begin with some developed knowledge of local road rules, add to this the individual components of the workings of the vehicle (mirrors, indicators, brakes, and accelerator), and then perhaps practice on quiet roads as they develop higher order driving proficiency. In our context, immersive simulation scenarios where participants are not equipped with the knowledgebase required to adequately engage—will often result in overwhelmed learners. We have all seen the participants that have been ‘scarred’ by previous poor simulation experiences, often due to a lack of consideration in this regard. Simulation faculty may often be guilty of this through our best-intentioned commitment to curate high levels of psychological fidelity without critical consideration of the toolkit required to interact with it.

To continue the illustration above, we would advocate that simulation designers closely consider the attributes and level of learner—building a simulation intervention for the learner rather than the other way around. This might look like a series of lectures or tutorials in the lead up to a simulation exercise, or perhaps exposing first-time learners to a scenario that feels less psychologically immersive. The benefit of curating environments that afford cognitive capacity within the working memory of learners allow them to make sense and build the scripts and schemas required for meaningful learning [25].

## The Scottish Simulation ‘KSDP’ Design Framework

To sense-make, sequence, and embed effective and efficient simulation in real-world clinical environments, we sought to formulate a simple and practical simulation design framework. The framework follows a ‘Knowledge’, ‘Skills’, ‘Drills’, and ‘Performance’ pathway (or KSDP). This pathway acknowledges that learning is multifaceted and complex, and it shapes the design of the simulation programme approach to four separate, sequenced domains:

- **Knowledge:** This domain speaks to the essential and desired intended learning outcomes that would most effectively be understood theoretically or empirically. This domain may most efficiently be explored in a workshop, online, or classroom setting. Simulation may be an appropriate tool but is often a less efficient way of exploring purely knowledge-based outcomes.
- **Skills:** This domain concerns the essential and desired learning outcomes that require practical performance or execution in some way, and often some practice and rehearsal. This takes a range of forms, from practising elements of communication techniques to inserting an intravenous cannula. It need not be a fully immersive scenario-based simulation exercise, but rather requires the learner to rehearse repeatedly and reflect on their actions through the principles of deliberate practice [26]. This domain is clearly easy to conceptualise for psychomotor tasks but is equally appropriate when considering cognitive skills. For example, the skill of data assimilation in preparation for a clinical handover. Whilst this domain may certainly utilise simulation-based methodology and technology to achieve its aims, the approach may look more like a task trainer and some coaching and less frequently require an expensive manikin or immersive simulation scenario to achieve optimal outcomes.
- **Drills:** Drills may be understood here as the stacking and sequencing of a collection of knowledge and skills which may be required to address intended learning outcomes through practice and rehearsal. Drills (such as the Advanced Life Support algorithm or utilisation of the ‘SPIKES’ (that is ‘Setting up the interview’, ‘Assessing the patient’s Perception’, ‘Obtaining the patient’s Invitation’, ‘Giving Knowledge and information to the patient’, ‘Addressing the patient’s Emotions with an empathic response’, and ‘Strategy and summary’) model in the breaking of bad news [27]) can be an effective and efficient method of increasing sets of skills in terms of efficiency, accuracy, and rate of completion of a task. In a similar way to the skills domain outlined above, running drill-based training need not require an immersive simulation scenario environment to be optimally effective, and might be better deployed in other contexts, such as a simulation-based mastery model, a ‘bootcamp’, or time trial [1]. However, this phase should not be limited to existing published algorithms and drills. The power of the approach is in seeing any clinical situation or problem in the context of an ordered set of steps, and then supporting learners to embed these steps in a meaningful way.
- **Performance:** The Performance aspect of the framework is the ‘bringing together’ of the Knowledge, Skills, and Drills domains into an often team-based, realistic and immersive environment where multiple performance aspects can be rehearsed and explored. This domain is a highly effective, safe, and efficient way to optimise individual and team performance and reflect upon the various team-based communication and behavioural elements. This step of the framework is often required to deliver the additional intended learning outcomes which are associated with the complexity in our health care environment. These outcomes are often more nuanced in nature, further exploring behaviours within teamwork, communication, or situational awareness for example—and optimally delivered in the context of immersive simulation. These outcomes should certainly be explored within the first three domains of the framework prior to this; however, it is the fourth and final domain that can be seen to consolidate this learning. Conversely, where the other domains are used effectively to meet the learning needs, it may be that this final performance domain is not required at all.

**Fig. 3:**
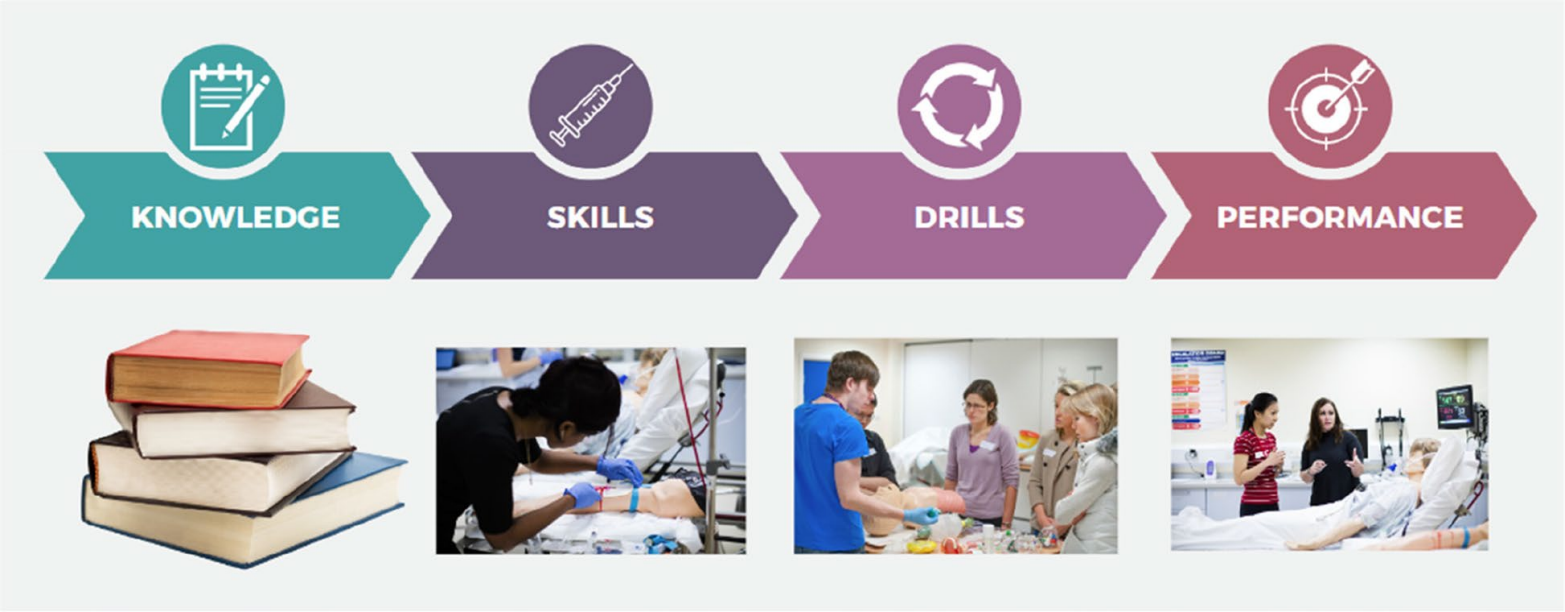
The Knowledge, Skills, Drills, Performance (KSDP) Framework

Through probing and exploring the nature of the request for a simulation-based programme (that is, sense-making), and then coherently arranging the educational interventions in a stepwise way (that is, sequencing), we ensure a more efficient and effective learning package is developed for the learners we design for (Fig. 3).

As a final illustration of how the framework might be implemented, we return to the case study [28].


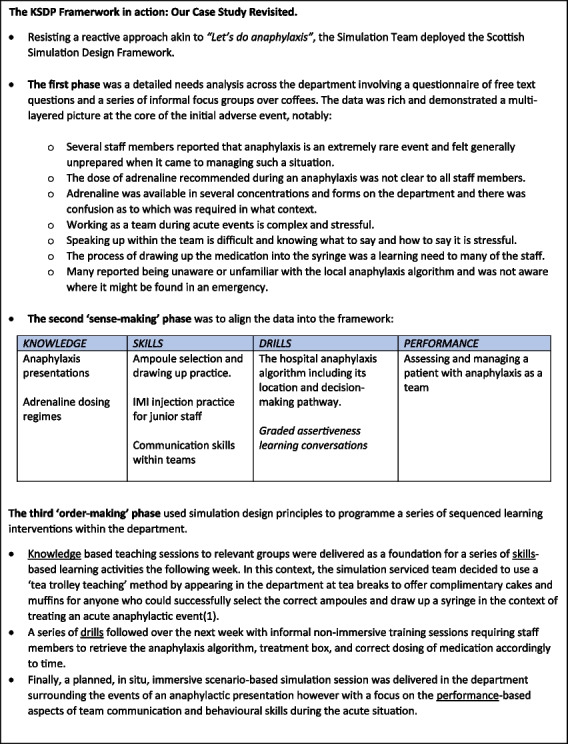


## Conclusion

The Scottish Simulation Design Framework is a sense-making and resource-aligning framework that has formed a foundation of many conversations in simulation programme design and simulation faculty development in our regional network. The design is simple and does not seek to make the claim to be all encompassing in addressing every circumstance or simulation-based technology or approach. However, the framework responds well to the complexity inherent in responding to the multifactorial nature of clinical teams and stands to promote a radically different execution and impact experience for many simulation designers, of which to hang their simulation methodology from.

## Data Availability

Not applicable.

## Abbreviation

KSDP: Knowledge, Skills, Drills, Performance
